# Heterogeneity of the Head and Neck Squamous Cell Carcinoma Immune Landscape and Its Impact on Immunotherapy

**DOI:** 10.3389/fcell.2019.00052

**Published:** 2019-04-09

**Authors:** Madison Canning, Gang Guo, Miao Yu, Calvin Myint, Michael W. Groves, James Kenneth Byrd, Yan Cui

**Affiliations:** ^1^Medical College of Georgia, Augusta University, Augusta, GA, United States; ^2^Department of Biochemistry and Molecular Biology, Georgia Cancer Center, School of Medicine, Augusta University, Augusta, GA, United States; ^3^Department of Otolaryngology, Georgia Cancer Center, School of Medicine, Augusta University, Augusta, GA, United States

**Keywords:** head and neck squamous cell carcinomas, heterogeneity, immune landscape, immunosurveillance, immunosuppression, neoantigen, checkpoint blockade

## Abstract

Head and neck squamous cell carcinomas (HNSCCs) are highly aggressive, multi-factorial tumors in the upper aerodigestive tract affecting more than half a million patients worldwide each year. Alcohol, tobacco, and human papillomavirus (HPV) infection are well known causative factors for HNSCCs. Current treatment options for HNSCCs are surgery, radiotherapy, chemotherapy, or combinatorial remedies. Over the past decade, despite the marked improvement in clinical outcome of many tumor types, the overall 5-year survival rate of HNSCCs remained ∼40–50% largely due to poor availability of effective therapeutic options for HNSCC patients with recurrent disease. Therefore, there is an urgent and unmet need for the identification of specific molecular signatures that better predict the clinical outcomes and markers that serve as better therapeutic targets. With recent technological advances in genomic and epigenetic analyses, our knowledge of HNSCC molecular characteristics and classification has been greatly enriched. Clinical and genomic meta-analysis of multicohort HNSCC gene expression profile has clearly demonstrated that HPV^+^ and HPV^-^ HNSCCs are not only derived from tissues of different anatomical regions, but also present with different mutation profiles, molecular characteristics, immune landscapes, and clinical prognosis. Here, we briefly review our current understanding of the biology, molecular profile, and immunological landscape of the HPV^+^ and HPV^-^ HNSCCs with an emphasis on the diversity and heterogeneity of HNSCC clinicopathology and therapeutic responses. After a review of recent advances and specific challenges for effective immunotherapy of HNSCCs, we then conclude with a discussion on the need to further enhance our understanding of the unique characteristics of HNSCC heterogeneity and the plasticity of immune landscape. Increased knowledge regarding the immunological characteristics of HPV^+^ and HPV^-^ HNSCCs would improve therapeutic targeting and immunotherapy strategies for different subtypes of HNSCCs.

## Introduction

Head and neck squamous cell carcinomas (HNSCCs) are epithelial tumors derived from mucosa linings of oral cavity, oropharynx, larynx, or hypopharynx. According to the recently published report GLOBOCAN 2018 (global cancer statistics) (Bray et al., 2018), more than 800,000 new HNSCC cases are diagnosed each year. Currently, the majority of head and neck cancers present with regionally advanced with lymph node metastases at the time of diagnosis. The patients are often given the standard treatment options of surgery, radiotherapy, chemotherapy, or a combination of these interventions, but 40–60% of treated patients experience recurrence and are unresponsive to subsequent therapeutic interventions (Haddad and Shin, 2008; Tolstonog and Simon, 2017). Therefore, despite the significant improvement in overall survival (OS) for patients with other tumor types, the 5-year OS rate of HNSCCs has not changed much over the past decade (Jemal et al., 2011; Torre et al., 2015; Bray et al., 2018). The classic causative factors for ∼80% of HNSCCs are heavy tobacco usage and/or excessive alcohol consumption (Haddad and Shin, 2008; Leemans et al., 2018). Due to a recent, substantial increase in human papillomavirus (HPV) infections in the Western world with a specific rise in the prevalence of HPV-positive oropharyngeal tumors in non-smokers, HPV-infection has emerged as another carcinogenic factor of HNSCCs (D’Souza et al., 2007; Castellsague et al., 2016). HNSCCs are diverse and complex diseases manifesting high levels of inter- and intra-tumoral heterogeneity as well as disparities in therapeutic response irrespective of clinical stage. Therefore, a better understanding of HNSCC biology and identification of specific markers or signatures for clinical prognosis and therapeutic targets will be invaluable for adapting advanced, targeted interventions to improve outcomes of HNSCC treatment.

During the past decade, the tremendous advances in next-generation sequencing (NGS) and analyses of alterations in gene expression/rearrangements, including DNA copy number, somatic mutations, and promoter methylation, have led to an exponential gain of genomic and epigenetic information regarding HNSCC molecular characterization and landscape (Hammerman et al., 2015; Leemans et al., 2018). These advances, especially in the context of HNSCC carcinogenesis, clinicopathology, and immunotherapy interventions, have provided significant insight into the diverse molecular mechanism of HNSCC carcinogenesis, the unique characteristics and heterogeneity of the HNSCC tumor microenvironment (TME), and the diversity in clinical responses among HNSCC subtypes (Hammerman et al., 2015; Tonella et al., 2017; Leemans et al., 2018). This information, along with continued in-depth investigation and translation into targeted therapeutic strategies, will lead to significant improvement in clinical outcomes. Here, we first briefly discuss our current understanding of the genetic landscape and molecular characteristics of HNSCCs with an emphasis on the potential implication of the cellular and immunological pathways and heterogeneity, followed by a discussion of basic tumor immunology, antitumor immunity, and the immune landscape of the HNSCC TME. We then conclude with a discussion of the current and potential new strategies against effective therapeutic targets toward the highly heterogeneous and immunosuppressive HNSCCs.

## The Genomic Landscape and Moleculr Classification of HNSCCs

Conventional HNSCC classification and clinical management are mainly based on anatomic location, phenotype, and clinical stages, including the existence of tumor-node-metastasis (TNM) and the depth of tumor invasion (Brierley et al., 2016). Nevertheless, for most of the advanced HNSCCs with regional metastasis, histological and clinical-staging do not correlate with clinical responses or prognosis (Hammerman et al., 2015; The Cancer Genome Atlas Network [TCGA], 2015; Leemans et al., 2018). Recent technological advances in comprehensive and integrative genomic and epigenetic analyses have made it possible to identify specific molecular markers for targeted therapeutic strategies, which improve personalized treatment and predication of recurrence/metastasis and clinical prognosis (reviewed in Tonella et al., 2017; Leemans et al., 2018).

With a rapid rise of HPV-positive (+) cases in ∼20% of HNSCC patients in the Western world, an emerging topic relating HNSCC carcinogenesis, cellular, and molecular heterogeneity to a clinical presentation is the involvement of HPV (The Cancer Genome Atlas Network [TCGA], 2015; Agalliu et al., 2016; Leemans et al., 2018). Recently, compelling results clearly demonstrated that HPV (+) and HPV-negative (-) HNSCCs are distinct subtypes in regard to molecular signatures, clinical presentation, and responses to therapy. For instance, HPV-infections are more prevalent in tumors originated from oropharynx, especially in Caucasians (Table 1) (The Cancer Genome Atlas Network [TCGA], 2015; Ragin et al., 2016; Fakhry et al., 2017; Buckley et al., 2018; Leemans et al., 2018; Razzaghi et al., 2018). Interestingly, HPV (+) oropharyngeal squamous cell carcinomas (OPSCCs) manifested pathologically as large ovoid nuclei with minimal cytoplasm and reduced keratinization and were mostly located in the periphery of tumors surrounding the proliferating tumor cell clusters (Buckley et al., 2018). The HPV (+) status of these OPSCCs was found to be in association with a better overall clinical prognosis (Table 1) (Fakhry et al., 2008, 2017; Buckley et al., 2018; Leemans et al., 2018). In contrast, HPV (-) OPSCCs, which presented as well keratinized with large amounts of cytoplasm and distinct cell borders, were more closely linked to tobacco/alcohol use, found with higher incidence in Asians and African American populations, and more predictive of a poor clinical prognosis (Fakhry et al., 2008, 2017; Ragin et al., 2016; Bray et al., 2018; Buckley et al., 2018; Leemans et al., 2018). It is also noteworthy that the incidence of HNSCC in males is two to three times of that in females worldwide (Table 1) (The Cancer Genome Atlas Network [TCGA], 2015; Bray et al., 2018). Therefore, we will describe and discuss the molecular landscape of HPV (+) and HPV (-) HNSCCs separately whenever possible. On the other hand, most of the molecular classification studies were performed using total HNSCC specimens regardless of HPV statuses; therefore, we must describe the consensus classification with less emphasis on HPV status. Because the genomic landscape and molecular signatures of HNSCCs are not the focus of this review, we will only briefly describe the general observations regarding HNSCC biology, molecular signatures of different tumor subtypes, and carcinogenic drivers, which have a direct implication on the immune landscape and immunotherapy of HNSCCs.

**Table 1:** Causal, anatomical, gender, and racial diversities, clinicopathology, and survival of the HPV-positive and HPV-negative HNSCCs.

Characteristic	Total number	HPV^+^ (%)	HPV^-^ (%)	References
**Gender**				
Female	76	4 (5)	72 (95)	The Cancer Genome Atlas Network [TCGA], 2015
Male	203	32 (16)	171 (84)	
Female^∗^	52	28 (54)	24 (46)	Ang et al., 2010
Male^∗^	271	178 (66)	93 (34)	
**Race**				
Caucasian	242	34 (14)	208 (86)	The Cancer Genome Atlas Network [TCGA], 2015
Non-Caucasian	37	2 (5)	35 (95)	
Caucasian^∗^	278	190 (68)	88 (32)	Ang et al., 2010
Non-Caucasian^∗^	45	16 (36)	29 (64)	
**Anatomical location**				
Oropharynx	33	22 (67)	11 (33)	The Cancer Genome Atlas Network [TCGA], 2015
Oral cavity	172	12 (7)	160 (93)	
Larynx	72	1 (1)	71 (99)	
Hypopharynx	2	1 (50)	1 (50)	
**Smoking (pack years)**				
<20	15	14 (93)	1 (7)	Fakhry et al., 2008
>20	72	17 (24)	55 (76)	
**Alcohol history**				
No alcohol use	85	5 (6)	80 (94)	The Cancer Genome Atlas Network [TCGA], 2015
Alcohol use	188	30 (16)	158 (84)	
**Overall survival probability**				
0–15 months	38(+)/58(-)	37 (97)	48 (83)	Fakhry et al., 2008
15–30 months	38(+)/58(-)	35 (92)	36 (62)	
60 months	36(+)/243(-)	(∼55)	(∼40)	The Cancer Genome Atlas Network [TCGA], 2015
**Relative survival^∗^**				
3-year overall survival^∗^	206(+)/117(-)	165 (82.4%)	51 (57.1%)	Ang et al., 2010
5-year overall survival^∗^	206(+)/117(-)	73 (35.4%)	22 (18.8%)	Ang et al., 2010

*^∗^Oropharyngeal SCC.*

### The Genomic Landscape of HPV-Positive HNSCCs

Human papillomavirus is a well-known causative factor for cervical cancers, associated with the 2008 Nobel Prize to Dr. Hausen (Hampton, 2008; Moody and Laimins, 2010). An increase in oropharyngeal tumors and their high prevalence of HPV-positivity (∼60%) implicated the potential causative effects of HPV for HNSCC malignancy (Chaturvedi et al., 2011; Castellsague et al., 2016). Similar to cervical cancers, HPV-16 was the most common subtype that accounted for ∼80% infected cases of the HPV (+) HNSCCs, determined by positive serological response to HPV-16 E6 protein, the *E6* and *E7* viral oncogene mRNA expression, or p16INK4a protein expression (Table 2) (Gillison et al., 2008; Shi et al., 2009; Ndiaye et al., 2014; Agalliu et al., 2016).

**Table 2:** Molecular landscapes that are impacted differentially in the HPV-positive and HPV-negative HNSCCs.

Gene	Prevalence	Mutation/alteration in function	Cellular process	References
**HPV^+^ HNSCCs**				
*E6* and *E7*	80%	Viral oncogene	Cellular transformation; functional inhibition of p53/RB1 proteins	Hammerman et al., 2015
*TP53/RB1*	Rare	Low mutation rate, functional inactivation	HPV-driven	Westra et al., 2008
*PIK3CA*	>50%	Amplification/mutation	AKT/mTOR pathway	Hammerman et al., 2015
*TRAF3*	8/14%	Truncation/recurrent deletion	Uncontrolled NF-KB signaling	The Cancer Genome Atlas Network [TCGA], 2015
*FGFR2/3*	>10%	Alteration/oncogene fusion (FGFR3-TACC3)	Activation of the RTK (receptor tyrosine kinase) pathway	Seiwert et al., 2015
*CD8, CD56, ICOS, LAG3, HLA-DR*	IMS subtype	Elevated levels of gene expression enhanced immune cell infiltration	CD8^+^ T and NK cell infiltration	The Cancer Genome Atlas Network [TCGA], 2015
**HPV^-^ HNSCCs**				
*TP53*	~86% common	Somatic mutations Chromosomal loss at 3p/17p	Tumor suppressor loss of function	The Cancer Genome Atlas Network [TCGA], 2015
		Copy number alteration	Loss of TP53 function	The Cancer Genome Atlas Network [TCGA], 2015
CDKN2A/RBI	Very common	Chromosomal loss at 9p	Tumor suppressor loss of function	The Cancer Genome Atlas Network [TCGA], 2015
*HRAS*	5–10%	Activating mutation	Constitutive activation of RAS pathway	Seiwert et al., 2015
*CASP8*	Co-occurrence with *HRAS* mutation	Inactivating mutation	Suppression of cell death	The Cancer Genome Atlas Network [TCGA], 2015
*EGFR, ERBB2, FGF1*	~30%	Amplification	Activation of the RTK pathway	The Cancer Genome Atlas Network [TCGA], 2015
*FAT1, AJUBA*,	Common	Inactivating mutation/deletion	WNT/b-catenin signaling	The Cancer Genome Atlas Network [TCGA], 2015
*N0TCH1*	Common	Mutation/deletion	Differentiation	The Cancer Genome Atlas Network [TCGA], 2015
*TP63*	Common	Gain of function	Differentiation	The Cancer Genome Atlas Network [TCGA], 2015
*NFE2L2, KEAP1*	~5%	Activating mutation	Oxidative stress	Hammerman et al., 2015

Because HPV-16 E6 and E7 viral proteins induce cellular transformation and prevent apoptosis via functionally inhibiting the activity of tumor suppressor p53 (TP53) and retinoblastoma 1 protein (RB1) (reviewed in Moody and Laimins, 2010), *TP53* and *RB1* gene mutations were rarely detected in HPV (+) HNSCCs (Table 2). Although some studies suggested an overall lower level of mutational loads in HPV (+) than in HPV (-) HNSCCs (Stransky et al., 2011; Hanna et al., 2018), others observed a comparable level of mutational burden or frequency, with differing profiles, between HPV (+) and HPV (-) HNSCCs (Hammerman et al., 2015; Seiwert et al., 2015; The Cancer Genome Atlas Network [TCGA], 2015). Nevertheless, the breadth of molecular alterations in HPV (+) HNSCCs were rather limited to the amplification of *PIK3CA* oncogene and/or *E2F1*, the truncation of TNF receptor-associated factor 3 (*TRAF3*), and the mutation and fusion of *FGFR2/3* gene (Table 2) (Stransky et al., 2011; Keck et al., 2015; Seiwert et al., 2015; The Cancer Genome Atlas Network [TCGA], 2015). Interestingly, a subset of the HPV (+) HNSCCs present with a distinct immune signature, including elevated levels of *CD8, CD56, ICOS, LAG3*, and *HLA-DR*, which is likely the result of an activated anti-viral (HPV) response (Table 2) (Keck et al., 2015; The Cancer Genome Atlas Network [TCGA], 2015; Hanna et al., 2017). In general, HPV (+) HNSCC patients have a significantly better prognosis of 5-year overall survival than that of HPV (-) patients (Ang et al., 2010; The Cancer Genome Atlas Network [TCGA], 2015; Fakhry et al., 2017).

### HPV-Negative HNSCCs

HPV (-) HNSCCs account for the majority of the cases with excessive smoking and alcohol usage as major risk factors (The Cancer Genome Atlas Network [TCGA], 2015; Leemans et al., 2018). This subtype of HNSCCs manifests with a wide variety of gene mutations, amplifications, and epigenetic alterations that are associated with increased metastases and worse clinical outcomes (Table 2) (Stransky et al., 2011; Keck et al., 2015; The Cancer Genome Atlas Network [TCGA], 2015; Leemans et al., 2018). One of the prominent molecular abnormalities of HPV (-) tumors is widespread loss-of-function mutations in the tumor suppressors *TP53* and *CDKN2A/RB1* or chromosomal loss at 9p (*CDKN2A*) and 3p and 17p (*TP53*) (Table 2). Other highly enriched molecular abnormalities found in HPV (-) tumors are *HRAS, CASP8*, amplification of receptor tyrosine kinase (RTK) genes and *PIK3CA* gene, and genes/pathways associated with WNT signaling (*FAT1* and *AJUBA*), squamous cell differentiation (*TP63, NOTCH1*, and *RIPK4*), and oxidative stress regulation (*NFE2L2* and *KEAP1*) (Table 2). Overall, HPV (-) HNSCCs exhibit diverse alterations in the gene expression profile driven by environmental carcinogenic factors that presents clinically with a high incidence of recurrence, metastasis, and poor response to conventional and advanced therapies (Stransky et al., 2011; Keck et al., 2015; The Cancer Genome Atlas Network [TCGA], 2015; Leemans et al., 2018).

### Molecular Classification and Heterogeneity of HNSCCs

Aside from HPV status, current consensus of molecular classification categorizes HNSCCs into classical (CL), basal (BA), mesenchymal (MS), and atypical (AT) subgroups, each with distinct gene expression profile and biological characteristics (Walter et al., 2013; The Cancer Genome Atlas Network [TCGA], 2015). Interestingly, these molecular subtypes exist across all anatomic sites and clinical stages, with the exception of hypopharyngeal cancers lacking the BA subgroup (Walter et al., 2013).

The CL subgroup is associated with increased levels of polyamine, cell proliferation, and genes involved in cell cycle regulation and metabolism pathways (Keck et al., 2015). The CL subgroup of HNSCCs have also been shown to express a relatively high level of *SOX2*, a gene responsible for maintaining the self-renewal of undifferentiated stem cells, as observed in squamous cell carcinoma of the lung and other tissues (Walter et al., 2013; The Cancer Genome Atlas Network [TCGA], 2015). In contrast, the BA subgroup is highly enriched for hypoxia-response genes, EGFR signaling associated genes, and *TP63*, exhibiting a signature of epithelial keratinization and differentiation (Walter et al., 2013; Keck et al., 2015; The Cancer Genome Atlas Network [TCGA], 2015).

The MS and AT subgroups are HNSCCs that consist of a higher frequency of HPV (+) tumors than the CL and BA subgroups (Walter et al., 2013; Keck et al., 2015; The Cancer Genome Atlas Network [TCGA], 2015). The consensus classification of HNSCCs categorizes most of the HPV (+) tumors into the AT subgroup, due to high expression levels of *RPA2* (*Replication Protein A2), E2F2*, and *SOX2* with a strong HPV signature, whereas only a limited number of HPV (+) tumors are classified into the MS subgroup (Walter et al., 2013; The Cancer Genome Atlas Network [TCGA], 2015). The MS subgroup is characterized as having an elevated expression of epithelial-to-mesenchymal-transition (EMT) associated genes, such as *DES* and *TWIST*, and mesenchymal cell-related genes, including *VIM* (vimentin), *MMP*s, *PDGFRA*, and *PDGFRB* (Walter et al., 2013; The Cancer Genome Atlas Network [TCGA], 2015). Differing from the classic subtype characteristics, a recent comprehensive and integrative study by Keck et al. (2015) using data from multiple HNSCC cohorts consisting over 900 patients revealed a strong presence of the MS-signature in some of the HPV (+) tumors. In addition to their MS-signature and downregulation of markers for epithelial differentiation and keratinization, this HPV (+) MS subgroup exhibited a distinct signature showing an elevated expression of immune genes, such as *CD8, ICOS, LAG3*, and *HLA-DRA*, which were defined as the inflamed/mesenchymal (IMS) subgroup (Keck et al., 2015). This observation agreed with earlier reports of a strong immune signature associated with elevated expression levels of CD56 and HLA class I in some of the HPV (+) HNSCCs within the AT subgroup (Walter et al., 2013). Interestingly, the clinical benefits of the elevated immune-signature seemed to be more prominent in HPV (+) HNSCC patients because no significant benefit in overall survival was observed in the HPV (-) IMS subgroup of HNSCC patients, agreeing with the differential immune cell profiles between the virally and non-virally infected individuals (Keck et al., 2015).

Together, these observations demonstrate that integrative genomic analyses in association with functional annotation provides valuable information for identifying molecular drivers of carcinogenesis, potential markers for prognosis, and HNSCC classification based on molecular signatures that may facilitate a better prediction for responsiveness to therapy. Importantly, it should be appreciated that the complexity and heterogeneity in the landscape of both HPV (-) and HPV (+) HNSCCs contribute to differential responses to therapeutic interventions, and thus should be thoughtfully considered when selecting the appropriate therapeutic targets and/or strategies.

## Immune Landscape of the HNSCC TME

**FIGURE 1:**
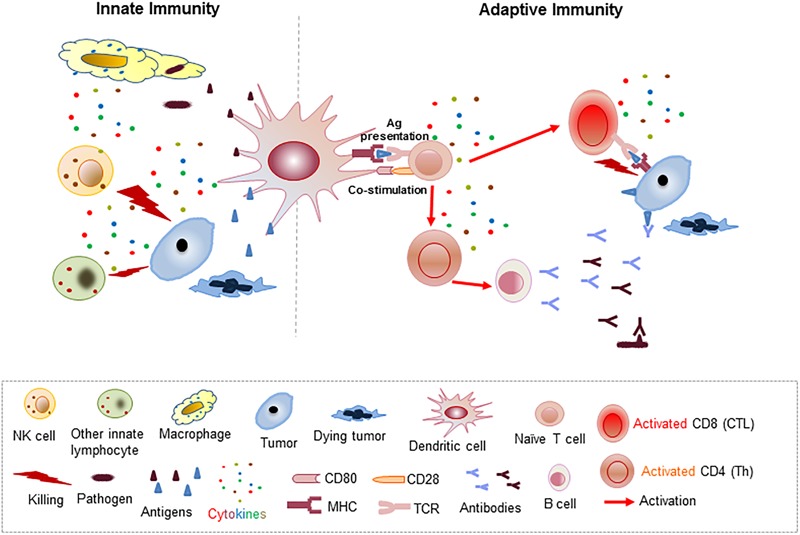
Schematic illustration of the cellular and molecular processes associated with innate and adaptive immunity mediated pathogen and tumor elimination.

**FIGURE 2:**
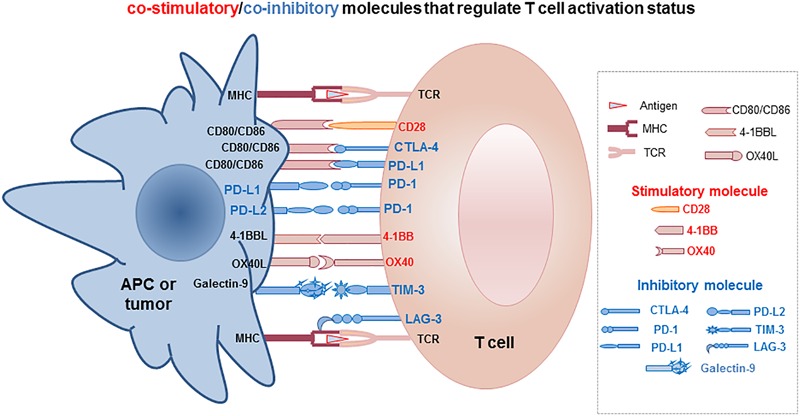
Schematic illustration of the co-stimulatory and co-inhibitory molecules involved in regulating T cell functional status of productive activation or tolerance/exhaustion.

The host immune system is an essential defense mechanism for recognizing and destroying pathogens, including bacteria, viruses, and other substances of foreign origin, via the coordinated and concerted activation of innate and adaptive immunity (Abbas and Janeway, 2000; Paul, 2013). The innate immune response is mediated through an acute mobilization and activation of macrophages, dendritic cells (DCs), and nature killer (NK) cells, which attack pathogens and tumors via endocytosis or cytolysis by cytokines or cytotoxic molecules in a non-antigen-specific manner (Figure 1). On the other hand, adaptive immunity involves activation of lymphocytes by activated antigen presenting cells (APCs), which present antigenic peptides through the MHC (major histocompatibility complex) surface proteins to T cells in the presence of co-stimulatory molecules such as CD28/CD80 (B7-1) or CD86 (B7-2) (Figure 1). Subsequently, activated CD4 T helper cells prompt the activation of cytolytic CD8 T cells and B cells for tumor and pathogen elimination in an antigen-specific manner (Figure 1). Under physiological conditions, immune activation is also associated with upregulation of immune inhibitory molecules, such as programmed death-1 (PD-1), PD-L1 (programmed death-ligand 1), and cytotoxic T-lymphocyte associated antigen (CTLA4). These inhibitory molecules are called checkpoint inhibitors, which serve as part of an intrinsic negative feedback loop to prevent sustained and uncontrollable T cell activation seen in self-destructive autoimmune diseases (Figure 2). Besides PD-1/PD-L1, CD28/CD80, CD86, and CTLA4, many more co-stimulatory and co-inhibitory molecules have been identified in the regulation of T cell activation and tolerance status during productive immunity and within the TME, respectively (Figure 2) (Chen and Flies, 2013; Chen and Mellman, 2013).

**FIGURE 3:**
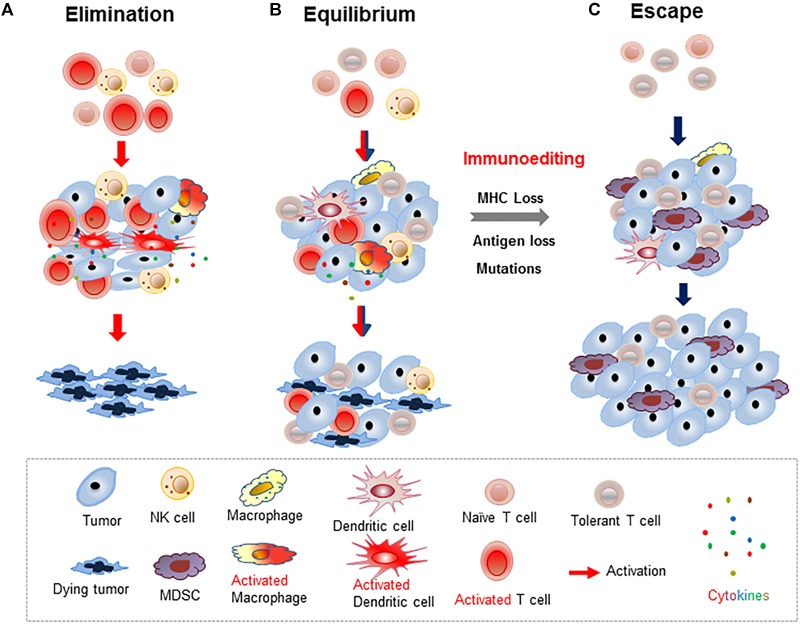
Schematic illustration of three outcomes of tumor immunosurveillance. **(A)** Tumors are eliminated by productive antitumor immunity of activated T cells and/or innate immune cells in the dominantly immunostimulatory TME. **(B)** Co-existence of activated immune cells that kill some of the tumor cells and ignorant/tolerant immune cells resulting in the survival of residual tumors and an overall tumor “dormancy.” **(C)** In the immunosuppressive TME, tumors and pro-inflammatory myeloid-derived suppressor cells (MDSCs) induce T cell tolerance and prevent tumors being recognized by host immunity, thereby promoting tumor progression.

It has long been debated whether recognition and elimination of cancer cells is an integral function of the immune system. In fact, as early as the 1900s, Paul Ehrlich speculated that a functional immune system could detect and control malignant tumors. This theory was further developed by Sir Frank Macfarlane Burnet and Lewis Thomas in the 1950s into the immunosurveillance hypothesis (Burnet, 1957, 1967; Thomas, 1982), which proposed the existence of an immunological mechanism for detecting and eliminating mutated abnormal cells. This hypothesis, however, had been challenged constantly until the late 1990s when compelling experimental evidence demonstrated the essential roles of immune effector molecules in suppressing tumor occurrence and progression. In the early 2000s, the cancer immunosurveillance hypothesis was further improved and refined by Robert Schreiber to accentuate the dynamic and bidirectional interactions between tumor cells and the immune system as a three-phase process, termed tumor “Elimination, Equilibrium, and Escape,” which describes the ongoing battle between the immune system and the tumor that determines tumor survival/growth and reshapes the tumor antigen pool and the immune landscape of the TME (Figure 3) (Dunn et al., 2002; Schreiber et al., 2011). Now, the immunosurveillance concept has been well appreciated to relay an important physiological process during carcinogenesis and tumor progression, and has provided invaluable insight into potential targets for immunotherapy intervention.

### Adaptive and Innate Immunity for Cancer Immunosurveillance

The first series of experimental evidence that firmly established the existence of immunosurveillance came from gene-targeted knockout mice with selective inactivation of key immune cytolytic molecules, such as perforin, interferon-γ (IFN-γ), and tumor necrosis factor-α (TNF-α), in either innate immune NK cells or adaptive immune cells (Dighe et al., 1994; van den Broek et al., 1996; Kaplan et al., 1998; Smyth et al., 2000a,b; Shankaran et al., 2001; Takeda et al., 2001). These genetically engineered mice lacking cytolytic function in their immune effectors were more susceptible to spontaneous or chemical-induced tumorigenesis. Likewise, mice with defective recombination-activating gene 2 (RAG2), which leads to T and B cell deficiency, were also more susceptible to spontaneous and carcinogen-induced tumorigenesis (Shankaran et al., 2001). Similarly, mice depleted of NK or NKT cells also showed an increased incidence of tumor development (Smyth et al., 2000a; Girardi et al., 2003), whereas mice treated with an NKT cell activation ligand during chemical-induced tumorigenesis manifested a reduced incidence and prolonged latency of tumor development (Hayakawa et al., 2003). Moreover, the observations that specific inactivation of classical T cells or γδ innate T cells via blocking TCR αβ or γδ chain rearrangement, respectively, led to an increased prevalence of tumorigenesis further supporting the role of both adaptive immunity and innate immunity in immunosurveillance (Girardi et al., 2003).

Clinically, it has long been noted that individuals with severe primary immunodeficiency or patients with vial or therapy-induced immunosuppression showed an increased risk of tumor development (Boshoff and Weiss, 2002; Oksenhendler et al., 2002; Salavoura et al., 2008; Engels et al., 2011). Moreover, in many types of human cancers, the number of tumor infiltrating CD3 or CD8 T cells is positively correlated with better clinical outcomes (Zhang et al., 2003; Galon et al., 2006; Hiraoka et al., 2006). Importantly, the observation that tumor antigen-specific T and B cells from tumor patients could be reactivated to induce tumor killing and regression provided direct clinical evidence of immunosurveillance. Thus, enhancing immunosurveillance and effector T cell infiltration to tumors is crucial for improving cancer immunotherapy.

### Cancer Immunoediting

Despite the existence of immunosurveillance and the observed elimination of some tumors by innate and adaptive immunity, a portion of tumor cells escaped elimination via mutations, alteration of MHC expression, or dysfunction of antigen processing machinery (APM) (Algarra et al., 2000; Marincola et al., 2000). This process was coined as “immunoediting” by Robert Schreiber (Dunn et al., 2002, 2004; Schreiber et al., 2011). Thus, active immunosurveillance imposes a selective pressure that “shapes” the immunogenicity of tumor cells and encourages/results in the escape of tumors that are less immunogenic via loss of tumor antigens and/or MHC expression (Figure 3). Gradually, the surviving tumors escape the immunosurveillance via accumulating mutations and are no longer recognized by the immune system. As such, cancer immunoediting is a dynamic process that encompasses tumor elimination, equilibrium, and escape (Figure 3) (Dunn et al., 2002; Schreiber et al., 2011). Spontaneous mutations have been detected in various tumors, which is attributed as one of the hallmarks of tumorigenesis and tumor recurrence (Hanahan and Weinberg, 2011; Schreiber et al., 2011; DuPage et al., 2012). Clinically, spontaneous or therapy-induced mutations that resulted in reduced tumor immunogenicity, including reduced MHC expression, have been frequently observed. Thus, throughout the tumorigenesis and tumor progression, there exists constant interaction between tumor and immune cells that affects the immunological status of activation vs. inhibition and thus, dictates the fate of tumor cells (Chen and Mellman, 2013).

### The Immunosuppressive TME of HNSCCs

Accumulating evidence suggests that tumor progression and metastases are markedly affected by the constituents surrounding and within the tumor parenchyma, the so-called TME. The TME is a highly complex, functional eco-system consisting of tumors and other cellular and molecular components. The cellular constituents of the TME consist of stromal cells [cancer-associated fibroblasts (CAFs), blood endothelial, and lymphatic endothelial cells], tumor-infiltrating lymphocytes (T cells, B cells, and NK cells), and myeloid populations [dendritic cells, macrophages, and myeloid-derived suppressor cells (MDSCs)]. Many of the tumor-infiltrating immune cells possess immune inhibitory function, such as regulatory T cells (Treg), MDSC, and type 2 macrophages (M2). At the molecular level, tumor-induced immunoevasion and immunosuppression are associated with downregulation of MHC molecules (human leukocyte antigen, HLA), inactivation of the APM, which prevents the processing and presentation of tumor antigens to CD8 T cells, and upregulation of the checkpoint inhibitors on tumors and immune cells (Ferris et al., 2006; Lopez-Albaitero et al., 2006). All of these cellular and molecular events actively and cooperatively enforce the immunosuppressive landscape (Biswas and Mantovani, 2010; Hanahan and Coussens, 2012; Curry et al., 2014).

Compelling evidence suggests that the immune landscape of HPV (+) HNSCCs differs from HPV (-) tumors in that the HPV (+) TME is associated with abundant immune infiltrates, whereas the HPV (-) TME incurs high mutational load. Currently, clinical treatment of HNSCC patients with either conventional chemo/radiotherapy regimens or the most recent advanced immunotherapy showed less favorable overall survival than patients with other tumor types receiving similar treatments, indicating the detrimental effects of an immunosuppressed HNSCC TME (Haddad and Shin, 2008; Curry et al., 2014; Schoenfeld, 2015; Tolstonog and Simon, 2017; Bray et al., 2018).

### Tumor Infiltrating Lymphocytes

Early clinical studies revealed the existence of T cell dysfunction, increased Tregs, and impaired NK cell activity, as well as an overall reduction in lymphocyte counts in HNSCC patients (Hoffmann et al., 2002; Reichert et al., 2002; Whiteside, 2005). Subsequent investigations illustrated that circulating and tumor infiltrating T lymphocytes from HNSCC patients exhibited abnormal signaling cascades, reduced proliferation, and spontaneous apoptosis (Hoffmann et al., 2002; Reichert et al., 2002). This observed T cell dysfunction is likely the result of an altered cytokine profile in the HNSCC TME, including increased IL-10, IL-6, and TGF-β secretion and reduced IL-12 levels (Lathers and Young, 2004; Lu et al., 2004; Sparano et al., 2004; Varilla et al., 2013). Moreover, there was an increase in Fas-ligand expression on HNSCCs and circulating vesicles, which also enhances the susceptibility of T cells to apoptosis (Gastman et al., 1999; Kim et al., 2005).

Interestingly, comprehensive clinical studies demonstrated that HPV (+) HNSCCs were among tumors with the highest immune infiltrates as compared to most other common tumor types (Mandal et al., 2016). Furthermore, these HPV (+) tumors showed high levels of Treg, Treg/CD8^+^, and CD56^dim^ NK cells, as well as an activated phenotype, including a significantly higher expression of CTLA4 (Mandal et al., 2016) and highly elevated PD-1 on T cells (Badoual et al., 2013) (Table 3). Remarkably, the increased levels of CD4^+^CD25^+^ Tregs and PD-1^+^ T cells correlated positively to better clinical prognosis in HNSCC patients (Badoual et al., 2006, 2013; Loose et al., 2008), which is different from the common observation of elevated Tregs in association with immunosuppression and poor clinical outcomes in other tumor types. It is proposed that these elevated CD4^+^CD25^+^ Tregs and PD-1^+^ T cells in HPV (+) HNSCCs indicate an on-going immunosurveillance against the HPV-viral proteins, which activate the negative feedback of suppressive mechanism. Consistent with this observation, a recent report also showed that in HPV (+) HNSCCs, CD3^+^ T cell infiltration was the highest when compared to other tumors, associated with a high frequency of CD56^+^CD3^+^ NKT cells and PD-1/TIM3 co-expressing CD8^+^ T cells (Hanna et al., 2017) (Table 3). Importantly, clinical and experimental observations suggest that the presence of IFN-γ cytokines, tumor infiltrating CD8 T cells, and PD-L1^+^ immune cells within the TME is likely an indication of pre-existing antitumor immune responses and is potentially more responsive to therapeutic interventions (Hegde et al., 2016).

**Table 3:** Immune landscapes of the HPV-positive and HPV-negative HNSCCs and clinical implications for targeted immunotherapy approaches.

	HPV (+) HNSCCs	HPV (-) HNSCCs	Reference
Overall tumor infiltrating lymphocytes	Relative high numbers		Mandal et al., 2016
		Low numbers	Hanna et al., 2017
Immune cells and phenotype	Increased CD4^+^CD25^+^ Tregs PD-1^+^ T cells and CD4^+^CD25^+^ Tregs		Loose et al., 2008; Badoual et al., 2013; Mandal et al., 2016
	High CD56^dim^ NK cells		Mandal et al., 2016
	Elevated PD-1 and CTLA4 on T cells		Mandal et al., 2016
	High CD56^+^CD3^+^ NKT cells		Hanna et al., 2017
	High PD-1^+^/TIM3^+^CD8^+^ T cells	Low PD-1^+^/TIM3^+^CD8+ cells	Hanna et al., 2017
Clinical prognosis/responsiveness			
Correlation between CD4^+^CD25^+^ Tregs and prognosis	Positive association	No correlation	Hanna et al., 2017
	Better clinical outcome	Poor outcome	Mandal et al., 2016
Overall immune landscape	Activated immune cell phenotype		Hanna et al., 2017
	Less immunosuppressive	Highly immunosuppressive	Hanna et al., 2017
Mutation load/dominate antigens	Low mutation load	High mutation load	Keck et al., 2015
	HPV-associated antigens	Neoantigens	The Cancer Genome Atlas Network [TCGA], 2015
Clinical responses to checkpoint blockade	Higher response rate	Low response rate	Seiwert et al., 2016
			Hanna et al., 2018
		Good response rate only in tumors with high mutation load and CD8 T cells	Hanna et al., 2018

In contrast, HPV (-) HNSCCs exhibited an overall reduced number of immune infiltrating cells, relatively low levels of CD8 T cells that co-expressed PD-1/TIM3, and lack of a positive association between CD4^+^CD25^+^ Tregs or PD-1^+^ T cells with clinical prognosis (Hanna et al., 2017) (Table 3). These HNSCCs exhibited a highly elevated smoking-related mutation profile and an unfavorable clinical outcome when compared to HPV (+) HNSCC patients (Mandal et al., 2016). These observations, together with those of HNSCC molecular landscape studies, further illustrated the high level of diversity and heterogeneity of the HNSCC TME, which can be affected by HPV status and potentially other unidentified factors, in their molecular and cellular profiles and clinical outcomes.

### Immunosuppressive Myeloid Cells

Myeloid cells, including granulocytes, monocytes, and their derivatives following activation or further differentiation (dendritic cells and macrophages) are crucial immune regulators and activators that bridge the innate and adaptive immunity under physiological conditions. Through antigen presentation and/or production of immune modulatory cytokines, myeloid cells induce either immune activation or tolerance (Dhodapkar et al., 2008; Biswas and Mantovani, 2010; Guilliams et al., 2014). During carcinogenesis and tumor progression, tumors also evade immunosurveillance by altering the myeloid cell phenotype and function, and creating a chronically inflamed immune landscape. In general, this tumor escape process can be mediated by active recruitment of MDSCs, macrophages, and macrophage polarization, and/or induction of regulatory DCs. The cellular and molecular alterations leading to tumor evasion can also be the result of altered antigen presentation capacity of these myeloid cells as well as enhanced production of immunosuppressive cytokines and metabolites (Dhodapkar et al., 2008; Biswas and Mantovani, 2010; Gabrilovich et al., 2012; Hanahan and Coussens, 2012; Elpek et al., 2014).

It has long been demonstrated that the HNSCC TME is associated with chronic inflammation and immune suppression (Whiteside, 2005), and expression of proinflammatory and proangiogenic cytokines (Chen et al., 1999; Lathers and Young, 2004; Sparano et al., 2004). This pro-inflammatory and immunosuppressive environment leads to the active recruitment of macrophages and MDSCs. In fact, two independent studies demonstrated that elevated CD68^+^ macrophages in HNSCCs were associated with clinical pathology and poor survival (Marcus et al., 2004; Wolf et al., 2015). Likewise, elevated MDSCs, characterized as CD11b^+^CD14^+^CD33^+^IL4Rα^+^HLA-DR^-^ cells, were also observed in the peripheral blood of HNSCC patients compared to that of healthy individuals (Chikamatsu et al., 2012). These MDSCs expressed elevated levels of CD86, PD-L1, and TGF-β, suppressed IFN-γ production and proliferation of activated T cells (Chikamatsu et al., 2012). Furthermore, treatment with neutralizing antibodies to block the effects of CD86, PD-L1, and TGF-β partially reversed the immunosuppressive function of MDSCs on T cell activation (Chikamatsu et al., 2012). Additionally, a DC maturation defect or differential maturation was observed in some HNSCC patients. Schuler et al. (2011) reported that monocyte-derived DCs from the peripheral blood of some HNSCC patients failed to mature in culture, implying an immunosuppressive environment in patients leading to defective APC maturation. In fact, a comparative study examining the abundancy and maturation status (CD83 expression) of S100^+^CD1a^+^ DCs in patients with oral squamous cell carcinoma (OSCC) showed that in primary tumors the total DC population was higher in patients without regional metastasis (PN-) than in patients with metastasis (PN+) (Kikuchi et al., 2002). On the other hand, the number of the CD83^+^ DC subpopulation was higher in tumors and draining lymph nodes of the PN+ patients than in the PN- patients (Kikuchi et al., 2002). These results further substantiate the high levels of diversity and heterogeneity in cellular phenotype, function, and location of myeloid subpopulations within the HNSCC TME.

### Cancer Associated Fibroblasts

Cancer-associated fibroblasts are specialized fibroblastic stroma representing the dominant non-hematopoietic cell type within the TME of many cancer types. CAFs are pivotal in tumorigenesis, tumor progression, chemoresistance, metastasis, and maintenance of cancer stem cells through their production of growth factors, chemokines, and extracellular matrix (ECM) (Bhowmick et al., 2004; Orimo et al., 2005; Turley et al., 2015; Gascard and Tlsty, 2016; Kalluri, 2016). Additionally, CAFs are actively involved in immune regulation by producing inflammatory cytokines/chemokines and soluble factors, and by directly interacting with immune cells to support the immune cell survival, function, and recruitment within the TME (Kraman et al., 2010; Liotta et al., 2015; Turley et al., 2015; Gascard and Tlsty, 2016; Kalluri, 2016). Experimental evidence suggests that CAFs are heterogeneous populations derived from various cell sources, including mesenchymal stem cells from the bone marrow, tissue resident fibroblasts, epithelial cells via EMT, fibrocytes, and likely other unidentified sources (Bhowmick et al., 2004; Orimo et al., 2005; Smith et al., 2013; Turley et al., 2015; Gascard and Tlsty, 2016; Kalluri, 2016). Clinical evidence clearly establishes the association between an increased CAF abundancy and poor prognosis in many tumor types (Turley et al., 2015; Gascard and Tlsty, 2016; Kalluri, 2016).

Within the highly heterogeneous HNSCCs, the existence of a MS-rich (presumably CAF-rich) subgroup has been revealed, which exhibits distinct molecular signatures and clinical presentation (Peng et al., 2011; Curry et al., 2014; De Cecco et al., 2015; Liotta et al., 2015; The Cancer Genome Atlas Network [TCGA], 2015; Tonella et al., 2017). Similar to the CAF markers used for other tumors, alpha-smooth muscle actin (α-SMA) was one of the commonly used and so far represents the most reliable marker for CAF-like cells in HNSCCs (Kawashiri et al., 2009; Lim et al., 2011; Marsh et al., 2011; Wheeler et al., 2014). Additionally, high expression levels of collagen and vimentin were also used for further confirmation of CAFs (Kawashiri et al., 2009). These HNSCC-CAFs expressed high levels of growth factors, cytokines/chemokines, and ECM (Kawashiri et al., 2009; Lim et al., 2011; Marsh et al., 2011; Wheeler et al., 2014), consistent with CAFs of other tumor types. Remarkably, a retrospective study of 282 cases of HNSCC specimens revealed a significant correlation of elevated α-SMA expression with poor overall survival regardless of the clinical stage (Marsh et al., 2011). Lim et al. (2011) also demonstrated that CAFs from genetically unstable HNSCCs (high mutational load and alterations in copy number or chromosomal loss) expressed significantly higher levels of α-SMA and integrin-α6 as compared to CAFs derived from genetically stable HNSCCs. Functionally, these α-SMA^+^ CAFs enhanced tumor progression, invasion, metastasis, glycolysis, persistence of cancer stem cells, and suppression of T cell activation and proliferation either by direct cell–cell interaction, production of soluble factors including TGF-β and hepatocyte growth factor (HGF), or elevated enzymatic activity of indoleamine 2,3-dioxygenase (IDO) (Knowles et al., 2009; Marsh et al., 2011; Wheeler et al., 2014; Liotta et al., 2015; Alvarez-Teijeiro et al., 2018).

Currently, our understanding of the CAF biology and CAF-tumor-immune cell interactions in the HNSCC TME is still limited. Given our knowledge of the heterogeneous cellular origins of CAFs, their co-evolution with, and likely co-regulation of the immune landscape during tumor progression, it is important and invaluable that more in-depth cellular and molecular investigations are performed for developing new targeted therapy. Along this line, a recent study investigated potential specific surface markers for HNSCC-CAFs because the currently used CAF markers are mostly intracellular proteins, which are not suitable for therapeutic targeting intervention. Through cDNA microarray analysis, Purcell et al. (2018) discovered a protein, leucine-rich repeat containing 15 (LRRC15), which is a membrane protein commonly expressed at high levels on mesenchymal cells, including CAFs in the HNSCC TME, but at low basal levels in healthy tissues. Similar to α-SMA expression, LRRC15 expression could be further upregulated by sustained exposure to TGF-β, which is one of the factors existing at high levels within the TME (Purcell et al., 2018). Therefore, LRRC15 represents a potential new immunotherapy target of CAFs. The implication of targeting LRRC5 in regard to releasing the immune suppression in the TME is yet to be tested.

### Heterogeneity of the HNSCC TME

Comprehensive and integrative genomic and epigenetic analyses demonstrate the extremely high heterogeneity in HNSCC molecular signature and landscape, whereas flow cytometry based assay provides additional evidence of the HNSCC heterogeneity in cellular phenotype, constituents of the TME, and immune landscape. On the other hand, recent compelling clinical evidence demonstrates that productive immunotherapy depends on not only the number of immune effectors in the TME, but also, and more importantly their accessibility to tumors (Chen and Mellman, 2013; Joyce and Fearon, 2015; Hegde et al., 2016). Specifically, three distinct patterns of T cell distribution in the TME were identified as immune inflamed, immune excluded, and immune desert (Joyce and Fearon, 2015; Hegde et al., 2016). In the immune inflamed tumors, T cells are heavily infiltrated into the solid tumors, whereas immune cells are primarily distributed in the peritumor region of the TME in the immune excluded tumors. The immune desert tumors manifest with a lack of immune cells in both the TME and at the peritumor region (Joyce and Fearon, 2015; Hegde et al., 2016). These distinct patterns of immune cell segregation in the TME are associated with the observed heterogeneous clinical responses and underscore the crucial contribution of direct effector-tumor interaction to the outcomes of immunotherapy (Joyce and Fearon, 2015; Hegde et al., 2016; Kather et al., 2018). Therefore, complementary information regarding the spatial distribution of immune cells and their potential interaction is as important as the cellular and molecular characterization of the tumor for proper design of targeted or individualized therapy.

### Heterogeneity of Immune Cell and CAF Composition in the TME

High levels of intertumor molecular and cellular heterogeneity of HNSCCs have been well documented and appreciated (Keck et al., 2015; The Cancer Genome Atlas Network [TCGA], 2015; Hanna et al., 2017). However, the intratumor heterogeneity, i.e., differential distribution of immune cells in different regions of the same tumor, remains largely unexplored. Recent studies employing multi-parameter flow cytometry analysis, especially with the simultaneous immunohistochemistry (IHC) staining-based topographic assessment of cancer-associated immune cell localization within the TME, support a high level of intratumor heterogeneity within certain HNSCC subgroups (Hanna et al., 2017; Kather et al., 2018). Importantly, this topographic assessment of immune cell spatial distribution in association with their cellular phenotype and functional analysis provides invaluable information for potential mechanistic explanation of the heterogeneous clinical responses and for identification of specific prognostic markers (Kather et al., 2018).

Similar to the recently published results of single-cell transcriptomic analysis of HNSCCs (Puram et al., 2017), our examination of human HNSCC specimens via multiplex IHC confirmed high levels of intratumor heterogeneity with variable prevalence of immune cell infiltration and compartmentalization (unpublished observation). Besides the observed differential immune cell segregation, CAF distribution and the expression of different CAF markers varied greatly within each HNSCC specimen. For instance, Puram et al. (2017) showed differential intra- and inter-tumor expression of podoplanin (PDPN) and fibroblast activation protein (FAP), both of which have shown to express on CAFs, in HNSCCs (Puram et al., 2017). Our multiplex IHC staining also reveal differential distribution of α-SMA and vimentin in HNSCC specimens in that vimentin^+^ cells appeared to co-localize with α-SMA^+^ cells within the TME, but only limited to a portion of α-SMA^+^ cellular structure (unpublished observation). The elevated vimentin expression in some α-SMA^+^ cells within the HNSCC TME is particularly interesting because it is an intermediate filament protein that is believed to be expressed during the EMT transition (Richardson et al., 2017). Nevertheless, additional cellular and molecular analyses are warranted before any conclusions are drawn concerning whether vimentin^+^ cells represent a transitional process from tumor cells to CAF-like cells via EMT or they represent a subtype of activated CAFs.

### Heterogeneity in Tumor Cell Phenotype and Mutational Burden

Despite the homogenous origin of HNSCCs from the mucosa epithelial linings in the upper aerodigestive tract, HNSCC tumors are known to be highly heterogeneous based on comprehensive genomic analyses (Curry et al., 2014; Hammerman et al., 2015; Schoenfeld, 2015; The Cancer Genome Atlas Network [TCGA], 2015; Ferris et al., 2016; Leemans et al., 2018). IHC analysis also demonstrates a heterogeneous loss of epithelial markers, including epithelial cell adhesion molecule (EpCAM) or Keratin 76 (Krt76), in clinical specimens of HNSCC or oral SCCs, respectively, leading to more aggressive tumor progression and altered immune landscape (Ambatipudi et al., 2013; Baumeister et al., 2018; Pan et al., 2018; Sequeira et al., 2018). Similarly, our multiplex IHC analysis also showed a heterogeneous loss of epithelial markers, including EpCAM and cytokeratin in HNSCC tumors examined as previously reported (Ambatipudi et al., 2013; Baumeister et al., 2018; Pan et al., 2018; Sequeira et al., 2018).

Another imperative and immunologically relevant heterogeneity of HNSCCs is the differential mutational load between HPV (+) and HPV (-) tumors (Keck et al., 2015; The Cancer Genome Atlas Network [TCGA], 2015). HPV (-) HNSCCs exhibit high levels of mutational burden with a wide spectrum of gene mutations and amplifications. Emerging evidence suggests that cancer cells harboring mutations acquire new tumor-associated antigens, termed “neoantigens.” Importantly, these neoantigens are perceived by host immune system as the “altered self” and therefore are ideal targets for cancer immunotherapy because of their exclusive expression in tumor cells (Schumacher and Schreiber, 2015; Wirth and Kühnel, 2017). In fact, experimental and clinical evidence strongly suggests that properly activated immune response against neoantigens is pivotal for the success of immunotherapy (Schumacher and Schreiber, 2015; Wirth and Kühnel, 2017).

Overall, HNSCCs exploit multiple immunosuppressive mechanisms to evade immunosurveillance and promote an immunosuppressive landscape that supports tumor initiation, progression, and metastasis. This high level of immunosuppression is further complicated by the heterogeneity at cellular, spatial, and molecular levels, all of which affect the clinical outcomes. Successful tumor elimination by immune cells largely depends on reversing/alleviating the immunosuppression and by the efficient access of activated anti-tumor effectors. Therefore, our better understanding of all aspects of the HNSCC heterogeneity will assist in the development of new immunotherapy strategies to improve the therapeutic outcomes (Chen and Mellman, 2013).

## Immunotherapy of HNSCCs: Cureent Status and Perspective

The goal of immunotherapy is to eliminate tumors or at least control tumor progression through strengthening immunosurveillance, enhancing the cytolytic activity of the immune effectors, and minimizing the potential of tumor equilibrium and escape. During the past decade, various immunotherapy approaches have been employed for HNSCC treatment. Although some of the immunotherapy regimens have resulted in an improvement of clinical outcome by prolonging cancer-free survival in a small fraction of patients when compared to the conventional therapy, the overall clinical response rate is lower than that observed in other tumor types treated with similar regimens. We will review the current status of the HNSCC immunotherapy trials that have mostly recruited patients with recurrent, metastatic (R/M) diseases regardless of their HPV-status. However, the clinical response of HPV (+) and HPV (-) patients are discussed separately whenever available.

### HNSCC Cancer Vaccines

Vaccines are extremely effective in protecting the human population from some of the deadliest infectious diseases and have contributed to the worldwide eradication of smallpox and restriction of polio and measles. Since the validation of the involvement of HPV in cervical cancers, effective HPV vaccines have been developed and employed globally in the high risk populations for prevention (prophylactic) of HPV-induced tumors. Over the past decade, HPV vaccines have been proven to be safe and highly effective in preventing HPV-associated cervical lesions (Schiller et al., 2012; Sabeena et al., 2018). The objective of prevention is to inhibit viral entry. Therefore, the immunization targets are mostly based on the L1 viral capsid proteins via viral-like particles, which stimulate a strong antibody response blocking viral entry and initial infection (Stanley et al., 2012).

Different from prophylactic vaccines, HPV-based therapeutic vaccines for cancer treatment rely heavily on productive activation of HPV antigen-specific T cells to target HPV-infected and transformed cells. For this purpose, HPV vaccines against viral oncoproteins E6/E7 have been developed for cervical cancers (Skeate et al., 2016), which represent an obvious immunotherapy option for HPV (+) HNSCC patients (Skeate et al., 2016; Schneider et al., 2018). This approach has been employed in a few HNSCC clinical trials using E6/E7 peptide, DNA, RNA, or attenuated vaccinia virus as the delivery vehicles. A systemic review of 11 independent HPV therapeutic vaccine trials from 2005 to 2017 revealed low therapy-associated toxicity in all 376 HNSCC patients with incurable, recurrent loco-regional, or distant metastatic disease at the time of enrollment (Schneider et al., 2018). Although not all of the trials were designed to demonstrate therapeutic efficacy, the clinical response rate for those with available data indicated that a range of 33–75% of patients showed a positive immune response, defined as elevated anti-HPV antibody, IFN-γ production, and T cell response (Schneider et al., 2018). Importantly, but not surprisingly, another clinical trial combining the PD-1 checkpoint blockade with therapeutic HPV vaccine demonstrated a further improvement in activating immune response to HPV-16 and prolonged overall survival as compared to either regimen alone (Massarelli et al., 2018). A recent report on a phase Ib/II clinical trial of HVP-specific DNA vaccine of 21 HNSCC patients also demonstrated an overall ~85% of the patients showed an increase in IFN-γ producing antigen-specific T cells that lasted longer than 1-year (Aggarwal et al., 2018). Some patients also manifested an elevated CD8^+^/Treg ratio and perforin-producing immune cells (Aggarwal et al., 2018) although the overall survival rate is not yet available. These results support the speculation that HPV (+) HNSCC patients respond better to immunotherapy, especially those that alleviate the existing immune suppressive elements in the TME.

For HPV (-) HNSCCs, high levels of mutational burden suggest the potential existence of targetable tumor-specific neoantigens for redirecting productive antitumor immunity (Gubin et al., 2015; Schumacher and Schreiber, 2015; Wirth and Kühnel, 2017). Because *TP53* mutation associated with accumulation of p53 protein represents one of the widespread gene alterations in the HPV (-) HNSCCs, targeting WT or mutant p53 via tumor vaccine has been a primary approach tested in clinical trials. An early report of a p53 and k-ras peptide vaccine trial demonstrated a response rate of ~42% HNSCC patients with an increased frequency of IFN-γ producing CTLs, associated with their prolonged survival (Carbone et al., 2005). The observations of Couch et al. (2007) further suggested that mutant p53 peptides bind to MHC molecules with higher affinity than wild-type p53 counterparts and activated p53-specific T cells in culture, thereby representing an effective target. Likewise, the recent results of a phase I trial of p53-peptide loaded autologous DC vaccine together with immune adjuvant demonstrated *in vivo* activation of p53-specicity T cells and a favorable 2-year disease-free survival with low levels of toxicity (Schuler et al., 2014). Associated with the increases in p53-specific CD8 T cells and elevated IFN-γ production, the frequency of Tregs were reduced in some patients (Schuler et al., 2014). Nevertheless, the authors concluded that stronger DC maturation stimuli are desired to further enhance/maintain DC function in the immunosuppressive TME of HNSCCs and to improve therapeutic efficacy (Schuler et al., 2014). Another phase II clinical trial of peptide-based vaccine against three antigens, LY6K, CDCA1, and IMP3, identified via cDNA microarray from HNSCCs demonstrated improved immune responses to these specific-antigens and furthermore, overall clinical outcome (Yoshitake et al., 2015).

In addition to the activation of conventional T cells, vaccines to activate invariant natural killer T (iNKT) cells were tested by Takami et al. (2018). iNKT cells are special types of T cells that recognize lipid antigens, such as α-galactosylceramide (α-GalCer), that present on CD1d. They are known to rapidly produce effector cytokines and orchestrate with other immune cells to fight against pathogens and cancers (reviewed in Bedard et al., 2017; Takami et al., 2018). The results of various clinical trials with HNSCC patients suggest that iNKT cells could be activated by α-GalCer-pulsed APCs *in vivo* and lead to antitumor immunity (Uchida et al., 2008; Kunii et al., 2009). Interestingly, these studies also demonstrated that the route and geolocation of APC delivery is important for immune activation because nasal submucosa delivery promoted antitumor immunity, whereas APC injection into submucosa of the oral floor led to immune tolerance induction (Uchida et al., 2008; Kunii et al., 2009; Kurosaki et al., 2011).

Overall, considering the relatively high level of either viral antigens or mutation-associated neoantigens and immune infiltrates in different subtypes of tumors, HNSCCs represent good candidates for immunotherapy, especially if the immunosuppressive elements are alleviated prior to or simultaneously, with the vaccine. Clinical translation of this strategy, especially for HPV (-) HNSCCs, may benefit from personalized immunotherapy, which employs identified/defined unique neoantigens from each patient or autologous tumor (lysate) vaccine.

### Adoptive Transfer (ACT) of Activated Tumor-Specific T Cells

Adoptive transfer of *ex vivo* activated and expanded autologous tumor antigen-specific T cells represents a promising strategy to obtain high number of productively activated effectors. Most of the T cells were activated and expanded *ex vivo* via cytokine and anti-CD3/CD28 or tumor specific-antigen-dependent stimulation followed by adoptive transfer to tumor patients. Alternatively, these autologous T cells can also be genetically engineered to recognize a defined antigenic epitope by incorporating a chimeric antigen receptor (CAR). So far, only limited cases of ACT for HNSCC treatment have been reported. In an early study, 15 HNSCC patients with recurrent and metastatic disease were treated with one dose of ACT of autologous T cells, which were obtained from draining lymph node and expanded *ex vivo* via mitogen stimulation (To et al., 2000). In this cohort of treated patients, three showed stable disease and two achieved favorable response, among which one experienced complete remission for 4+ years (To et al., 2000). Likewise, Jiang et al. (2015) reported the results of an ACT clinical application of *ex vivo* expanded autologous T cells by anti-CD3 and cytokine in a cohort of 43 HNSCC patients following their first line chemo- and/or radio-therapy treatment. Overall, a modest improvement in the median progression-free survival from 40 months in the non-ACT control group to 56 months in ACT treated patients. Additionally, 3-year overall survival to 58 months as compared to the non-ACT control of 45 months (Jiang et al., 2015). In a phase II trial, patients with EBV^+^ nasopharyngeal carcinomas were first treated with four cycles of chemotherapy followed by up to six does of EBV-specific T cells recognizing viral protein LMP2 (Chia et al., 2014). The overall 2-year and 3-year survival rates were ~63% and ~37%, respectively. Strikingly, five patients experienced a complete remission for longer than 34 months, and the overall immune response in this cohort of was ~71% (Chia et al., 2014).

Remarkably, one recent report of a personalized immunotherapy for HPV-associated cervical cancer via adoptive transfer of *ex vivo* activated autologous tumor-infiltrating T cells revealed that effective elimination of HPV-associated cancers was dependent on T cells specifically targeting mutant endogenous neoantigen and cancer germline antigens rather than viral antigens (Stevanović et al., 2017). Thus, it is speculated that a similar therapeutic strategy may be implemented for HPV-associated HNSCCs.

### Checkpoint Inhibitor Therapy

The immune landscape of HNSCCs, especially HPV (+) tumors, is associated with elevated expression of the checkpoint molecules PD-1 and/or CTLA-4 on T cells (Badoual et al., 2006; Loose et al., 2008; Badoual et al., 2013; Mandal et al., 2016). In a subset of HNSCC patients, PD-L1 expression is frequently observed on a variety of immune and non-immune cells, including CAFs and tumor cells (Concha-Benavente et al., 2016). Therefore, the PD-1/PD-L1 checkpoint pathway is highly active in the HNSCC TME and suppressing the checkpoint pathway, either as a monotherapy or in combination with other immunotherapy interventions, represents a promising target for enhancing anti-tumor responses to control and eliminate HNSCCs.

Early checkpoint inhibitor clinical trials for HNSCC treatment did not discriminate patients based on their HPV status and showed an overall response rate (ORR) of ~10–20% among the total treated HNSCC patients. As we now appreciate the high level of heterogeneity in the TME of HNSCCs concerning the HPV status and tumor types, it becomes clear that analyzing and presenting the HNSCC clinical trial results by segregating HPV (+) patients from HPV (-) cases will be more informative. For instance, Keynote 012 Phase 1b anti-PD-1 antibody (pembrolizumab) trial treated a cohort of 60 R/M HNSCC patients positive for PD-L1 expressing tumors (>1% via IHC staining), with 10 mg/kg every 2 weeks. The ORR for the entire cohort was 18%, specifically with a 25% ORR for HPV (+) patients and 14% for HPV (-) patients (Seiwert et al., 2016). An expansion of this trial involved another cohort of 132 HNSCC patients, regardless of HPV and PD-L1 status, receiving the same antibody, in a dose of 200 mg every 3 weeks, that demonstrated a similar ORR of 18–20%. Interestingly, ORR for PD-L1 positive patients was 22%, significantly higher than PD-L1 negative patients (4%) (Chow et al., 2016). A follow up report of the long-term effects confirmed a durable response and clinical benefits in these treated patients with a 12-month ORR of higher than 71%, survival rate of 38%, and even antitumor responses in some patients lasting for longer than 30 months (Chow et al., 2016; Seiwert et al., 2016; Mehra et al., 2018).

A similar phase 3 anti-PD-1 (nivolumab) trial, Checkmate 141, which enrolled 361 recurrent HNSCC patients who failed standard chemotherapy, treated the patients with either 3 mg/kg body weight of anti-PD-1 every 2 weeks or conventional single-agent systemic therapy. In patients receiving nivolumab, the ORR and 6-month/1-year survival rate were better than those who received standard single-agent therapy (Ferris et al., 2016; Harrington et al., 2017), and a follow up report of 2-year long-term survival indicated a prolonged survival benefit for patients with PD-L1 positive tumors over those with PD-L1 negative tumors, regardless of HPV status (Ferris et al., 2018a). On the other hand, a recent study of a cohort of 126 HNSCC patients treated with anti-PD-1/L1 therapy suggested that HPV (+) patients experienced better clinical responses and outcomes compared to HPV (-) patients (Hanna et al., 2018). Remarkably, HPV (-) patients whose tumors exhibited higher mutational load and CD8^+^ T cell infiltrates showed a better response to the checkpoint inhibitor therapy, whereas patients with CD8^+^ T cells manifesting an exhausted phenotype of TIM-3/LAG-3 co-expression with PD-1 were poor clinical responders to the checkpoint inhibitors (Hanna et al., 2018).

The demonstration of differential clinical responses to checkpoint inhibitors in patients with PD-L1^+^ tumors is remarkable. Early studies reported the co-existence of PD-1^+^ T cells and PD-L1^+^ tumors with CD68^+^ TAMs in HPV (+) HNSCCs (Lyford-Pike et al., 2013). Furthermore, the observed association of PD-L1^+^ tumors with CD8 T cell expression of PD-1 and significantly elevated IFN-γ mRNA within the same TME indicated that PD-L1 was upregulated by activated T cells in the TME to augment an immunosuppressive landscape by enforcing the PD-1/PD-L1 pathway (Lyford-Pike et al., 2013). Thus, PD-L1 positivity may be considered as a potential marker for clinical response of HPV (+) HNSCC patients to checkpoint inhibitors, although a recent study of 126 HNSCC patients treated with anti-PD-1/L1 therapy demonstrated that PD-L1 alone could not serve as a robust predictor of clinical response (Hanna et al., 2018). Nevertheless, the targeted therapy of anti-PD-L1 antibodies (durvalumab or atezolizumab) has been employed in clinical trials (Cavalieri et al., 2018; Colevas et al., 2018). Colevas et al. (2018) reported the results of a phase 1a trial of 32 HNSCC patients receiving anti-PD-L1 every 3 weeks, which showed an ORR of 22% and no clear differences between HPV (+) and HPV (-) patients. The results of other trials are yet to be reported (Cavalieri et al., 2018).

To determine whether simultaneous blockade of two independent checkpoint molecules of PD-1 and CTLA4 will further improve the clinical outcome, Schwab et al. (2018) tested the combination effects of nivolumab and ipilimumab (anti-CTLA4) and reported a clinical case concerning treatment of a refractory HNSCC patient. A near complete remission was observed within 5 months of the combinatory treatment. Furthermore, following the onset of a local relapse at 7 months, combined radiotherapy and anti-PD-1 regimen was able to control tumor progression and support survival of the patient in stable disease for longer than a year (Schwab et al., 2018).

### Other Strategies to Reverse Immunosuppression and Reactivate Antitumor Immunity

Therapeutic interventions to enhance or reactivate antitumor immunity can be achieved by either alleviating the immunosuppressive cellular subpopulations or activating co-stimulatory pathways. Although many of these approaches have been tested in experimental models, publicly accessible clinical data are limited. Currently, the results of a few HNSCC clinical studies associated with inhibiting/reducing the immune inhibitory myeloid populations, such as MDSCs, or enhancing the immunostimulatory pathways have been published.

Tadalafil is an inhibitor of phosphodiesterase 5 (PDE5), which suppresses the function of MDSCs by inhibiting the production of iNOS and arginase-1. Califano et al. (2015) reported the employment of Tadalafil as a neoadjuvant in a phase II clinical trial with a cohort of 40 HNSCC patients. Overall, Tadalafil treatment led to a significant reduction of MDSCs and Tregs in both circulation and tumors, as well as an elevation of circulating CD8 T cells and improved T cell proliferative capacity *in vitro* (Califano et al., 2015). In a similar clinical study with 35 HNSCC patients, Weed et al. (2015) showed that Tadalafil modified the immune landscape of the TME with a significant increase in intratumor CD69^+^CD8^+^ T cells and a concordant reduction in Tregs, following a dose-dependent pattern. Because the objective and endpoint of these studies are immunomodulation, not the clinical improvement of tumor progression or patient survival, the long-term effects of Tadalafil treatment on HNSCC patient survival is unknown.

Anti-EGFR antibody, cetuximab, is a standard FDA approved targeted agent for HNSCC treatment. Currently cetuximab alone, or in combination with conventional radio- or chemo-therapy, only provides temporary and modest clinical benefit (Leemans et al., 2018; Zandberg and Ferris, 2018). Recently, the potential of cetuximab as a neoadjuvant for immune modulation has been evaluated. In a phase 1b clinical trial with 14 HNSCC patients enrolled, cetuximab together with a TLR8 agonist, motolimod, reversed MDSC-induced immunosuppression by inducing their conversion into M1 macrophages and improved antitumor immunity associated with increased number and function of tumor infiltrating CD8 T cells (Shayan et al., 2018). The results of an extended clinical study of a cohort of 195 R/M HNSCC patients treated with cetuximab and TLR8 agonist also demonstrated a T cell profile of immune activation and observed significant improvement in immune response at the injection site, especially in HPV (+) patients (Ferris et al., 2018b). However, no significant improvement in either progression-free survival or overall survival was reached (Ferris et al., 2018b). Mechanistic studies of cetuximab-induced immune modulation, either cetuximab alone or in combination with anti-CD137, illustrated that cetuximab activates NKs and DCs via Fc receptor-dependent pathway, subsequently leading to the activation of Th1/CTL responses and elevated APM for activation of tumor-specific T cells (Srivastava et al., 2017).

## Perspectives

Recent major advances in cancer immunotherapy, especially the immune checkpoint inhibitors targeting the PD-1/PD-L1 and CTLA-4 pathways, demonstrate remarkable curative benefits for some cancer patients. Despite a relatively low clinical response rate of HNSCC patients to the checkpoint inhibitors, the above described HNSCC clinical trial results with molecular and cellular profiles resulting from the above described HNSCC clinical trials provide invaluable insight into the challenges and opportunities for further improving the clinical outcomes of HNSCC immunotherapy.

It is now clear that HPV (+) HNSCCs are more responsive to immunotherapies, including the checkpoint inhibitor therapy, than HPV (-) tumors and associated with a better clinical prognosis. Notably, the immune landscape of HPV (+) HNSCCs exhibits a unique profile of inflamed, yet immunosuppressed, TME with heavy immune infiltrates of CD8^+^PD-1^+^ T cells and Tregs. This information suggests that HPV (+) tumor-associated immune infiltrates are more likely to respond to immune activation stimuli when the existing immune suppressive elements are timely removed/eliminated. To this end, Treg depletion or in combination with a checkpoint inhibitor, is likely more productive for immune activation than either regimen alone. Thus, it is proposed that upon depletion or inhibition of the immunosuppressive elements, tumor-specific T cells can be productively activated by professional APCs. This active regimen of T cell activation can be achieved via DC or tumor vaccines, as well as ACT generated against either HPV-specific antigens or tumor-specific neoantigens. Given the recent report of autologous neoantigen-specific T cell-mediated effective elimination of HPV-associated cervical cancers (Stevanović et al., 2017), it is anticipated that similar therapeutic effects can be achieved for treating HPV (+) HNSCCs. Different from the HPV-viral antigen-specific T cell activation, the neoantigen-specific antitumor immunity relies on individualized immunotherapy maneuvers because the mutation events and corresponding neoantigens that vary among patients, but support more productive antitumor immunity with better clinical outcomes.

The TME of HPV (-) HNSCC is highly immunosuppressed and associated with low levels of immune infiltrates. Existing clinical data suggest that HPV (-) HNSCCs are poor responders to immunotherapy, including the checkpoint inhibitor therapy, that is likely due to their immune excluded or desert landscape. Nevertheless, besides the overall lack of T cell accessibility to tumors, this observed unresponsiveness to immunotherapy can also be the result of the enforced immunosuppression by immune inhibitory cytokines/molecules, APM dysfunction, and/or immunosuppressive myeloid populations. Given the highly heterogeneous nature of the HNSCC TME, it is imperative to examine the pattern of immune cell distribution within the TME via IHC-based topography. In combination with the flow cytometry based cellular profiling and genomic based molecular profiling, IHC topographic results will assist in identifying the specific immunosuppressive pathway(s) or element(s) as targets for the individualized immunotherapy strategy to reverse the immune suppression and simultaneously promote neoantigen specific-antitumor immunity. The existence of widespread high levels of mutation burden in the HPV (-) HNSCC tumors present a favorable opportunity for activating a broad scope of neoantigen-specific antitumor immunity when the dominant immunosuppressive mechanism in the HNSCC TME is identified and alleviated. Routine clinical protocols for targeted MDSC or Treg depletion or conversion of M2 macrophages to activated DCs/M1 macrophages are established for many tumor types and can be employed for HNSCCs. It is speculated that the more challenging aspect of a productive strategy for eliminating HPV (-) HNSCC tumors is to enhance T cell accessibility to tumors. This may be improved by the checkpoint blockade in combination with the administration of specific chemokines that improve T cell mobility, such as CXCL9, CXCL10, and CXCL11. Furthermore, the defective or dysfunctional APM in the TME can be addressed by cytokine-induced HLA upregulation and the employment of NK-based tumor elimination.

Besides the well studied immunosuppressive cellular subsets of MDSCs, Tregs, and M2 macrophages, CAFs represent another crucial population that not only provides structure stability for the TME, but also promotes tumor survival/metastasis and regulates the immune landscape of the TME. Therapeutic interventions specifically targeting CAFs represent an appealing multipronged strategy that reduces the tumor survival factors and reverses the immunosuppressive landscape, thereby enhancing antitumor immunity and improving therapeutic outcomes. On the other hand, our understanding of HNSCC-CAF immunobiology and the specific surface markers for therapeutic targeting is still limited. One of the surface molecule LRRC15, identified by Purcell et al. (2018) represents an attractive candidate for further exploration toward its potential clinical application of targeting HNSCC-CAFs.

## Conclusion

In conclusion, recent clinical, genomic, and cellular studies of HNSCCs demonstrate the high levels of heterogeneity and immunosuppression in the HNSCC TME. The differential molecular and immune landscapes between HPV (+) and HPV (-) tumors present new opportunities for the development of individualized targeted immunotherapy strategy. It is proposed that the informed design of immunotherapy trials based on our understanding of HNSCC biology, molecular and immunological landscape, as well as topography of immune cell distribution in the TME, will assist in developing new strategies for a productive antitumor immunity to improve the clinical outcomes.

## Author Contributions

MC, GG, and YC reviewed the literature and wrote the manuscript. MY, CM, MG, and JKB reviewed the literature, participated in discussion, and revised the manuscript.

## Conflict of Interest Statement

The authors declare that the research was conducted in the absence of any commercial or financial relationships that could be construed as a potential conflict of interest.
